# Promising Potential of Crude Polysaccharides from *Sparassis crispa* against Colon Cancer: An In Vitro Study

**DOI:** 10.3390/nu13010161

**Published:** 2021-01-06

**Authors:** Natalia Nowacka-Jechalke, Renata Nowak, Marta Kinga Lemieszek, Wojciech Rzeski, Urszula Gawlik-Dziki, Nikola Szpakowska, Zbigniew Kaczyński

**Affiliations:** 1 Department of Pharmaceutical Botany, Medical University of Lublin, 1 Chodźki Street, 20-093 Lublin, Poland; renata.nowak@umlub.pl; 2 Department of Medical Biology, Institute of Rural Health, 2 Jaczewskiego Street, 20-090 Lublin, Poland; martalemieszek@gmail.com (M.K.L.); rzeski.wojciech@imw.lublin.pl (W.R.); 3 Department of Functional Anatomy and Cytobiology, Maria Curie Skłodowska University, 19 Akademicka Street, 20-033 Lublin, Poland; 4 Department of Biochemistry and Food Chemistry, University of Life Sciences in Lublin, 8 Skromna Street, 20-704 Lublin, Poland; urszula.gawlik@up.lublin.pl; 5 Faculty of Chemistry, University of Gdańsk, 63 Wita Stwosza Street, 80-308 Gdańsk, Poland; nikola.szpakowska@ug.edu.pl (N.S.); zbigniew.kaczynski@ug.edu.pl (Z.K.)

**Keywords:** β-glucan, anticancer activity, antioxidant, anti-inflammatory, cyclooxygenase, lipoxygenase, cauliflower mushroom

## Abstract

The aim of the present study was to evaluate in vitro the beneficial potential of crude polysaccharides from *S. crispa* (CPS) in one of the most common cancer types—colon cancer. The determination of the chemical composition of CPS has revealed that it contains mostly carbohydrates, while proteins or phenolics are present only in trace amounts. ^1^H NMR and GC–MS methods were used for the structural analysis of CPS. Biological activity including anticancer, anti-inflammatory and antioxidant properties of CPS was investigated. CPS was found to be non-toxic to normal human colon epithelial CCD841 CoN cells. Simultaneously, they destroyed membrane integrity as well as inhibited the proliferation of human colon cancer cell lines: Caco-2, LS180 and HT-29. Antioxidant activity was determined by various methods and revealed the moderate potential of CPS. The enzymatic assays revealed no influence of CPS on xanthine oxidase and the inhibition of catalase activity. Moreover, pro-inflammatory enzymes such as cyclooxygenase-2 or lipooxygenase were inhibited by CPS. Therefore, it may be suggested that *S. crispa* is a valuable part of the regular human diet, which may contribute to a reduction in the risk of colon cancer, and possess promising activities encouraging further studies regarding its potential use as chemopreventive and therapeutic agent in more invasive stages of this type of cancer.

## 1. Introduction

Polysaccharides constitute an abundant group of macromolecules present in fungal cell walls. Due to their wide structural variability, they have been shown to have great potential to be biological response modifiers (BRMs). The composition of monosaccharide residues, including their sequence and placement as well as their connections and position of glycosidic linkages, affect polysaccharide activities [1]. One of the mushroom polysaccharides—chitin—is a water-insoluble and indigestible compound in the human gastrointestinal tract acting as dietary fiber [2]. In turn, most polysaccharides present in mushrooms are water-soluble glucans with different types of glycosidic linkages, e.g., (1→3)-α-glucans and (1→3), (1→6)-β-glucans [3].

Mushrooms constitute an inexpensive and abundant source of glucans with health-promoting potential. Nowadays, when the number of health-conscious consumers is growing, there is a need for developing new strategies for the acquisition of beneficial glucans. According to the latest data, the global market of β-glucans is expected to grow significantly and reach over 1 billion dollars in 2020 [4].

Mushroom polysaccharides, especially glucans, are known to exert anticancer activity through immunostimulatory potential, which involves the activation of the innate immune system as well as the acceleration of the host’s defense mechanisms [1,5]. In addition to the anticancer potential, mushroom polysaccharides possess a vast spectrum of biological activities, including antimicrobial, antiviral, antioxidant, anti-inflammatory, or prebiotic properties [6,7,8,9,10]. The major glucans isolated from the fungal species used in the treatment of cancer in Asian countries are β-glucans, e.g., lentinan from *Lentinus edodes*, schizophyllan from *Schizophyllum commune*, krestin from *Trametes versicolor*, and grifolan from *Grifola frondosa* [11,12,13,14]. Colon cancer is believed to be preventable with the use of natural chemopreventive agents. The strategy of chemoprevention assumes reversing, suppressing, or preventing carcinogenic progression [15]. With their multidirectional activity, mushroom polysaccharides are able to act at different steps in the carcinogenic process, with the overall goal of reducing cancer incidence. Moreover, there are some already known strategies based on the use of mushroom β-glucans not only to inhibit tumor growth but also induce synergistic effects with chemotherapeutic agents or other immune stimulators. An innovative approach assumes that β-glucans may be used to deliver nanoparticles containing chemotherapeutic agents to the colon cancer site to improve their therapeutic efficacy [16].

Moreover, there are many applications of β-glucans in food products related to their ability to form a gel and enhance the viscosity of aqueous solutions. The use of β-glucans allows the replacement of fat to develop calorie-reduced food products or enhance their appearance and texture [17]. Therefore, they can be considered functional food ingredients providing consumers with health benefits and bringing significant technological advantages.

*Sparassis crispa*, also known as cauliflower mushroom in English or Hanabiratake in Japanese and Ggoksongee (meaning a blossom) in Korean, is an edible and medicinal mushroom growing in the temperate regions of Europe and North America [18]. It is also a very popular cultivable species in Asian countries, especially in Japan [19]. Despite its popularity, bioactive polysaccharides from *S. crispa* have not been exactly defined and studied to date. The present study is an attempt to extend this knowledge. The aim of the present study was to evaluate the content of α- and β-glucans and determine the chemical composition and structure of the crude polysaccharides from *S. crispa* collected from the natural environment in Poland. Furthermore, the anticancer, anti-inflammatory and antioxidant activity of *S. crispa* crude polysaccharides were examined in vitro to determine their chemopreventive potential.

## 2. Materials and Methods

### 2.1. Materials

The wild-growing fruiting bodies of *Sparassis crispa* (Wulf.: Fr.). were collected in the forests of Puszcza Solska (Lublin Voivodeship, Poland) in September 2018. Mushroom specimens were authenticated by Prof. Renata Nowak from the Chair and Department of Pharmaceutical Botany, Medical University of Lublin, Poland (voucher specimen No. MSH-076). After collection, the mushrooms were immediately lyophilized, pulverized and kept in a freezer (−30 °C) until further analysis.

### 2.2. Chemicals and Apparatus

The COX (ovine) Colorimetric Inhibitor Screening Assay Kit was obtained from Cayman Chemical Company, Ann Arbor, MI, USA. The Megazyme Mushroom and Yeast Beta-Glucan Assay Kit was purchased from Megazyme International Ireland Ltd., Wicklow, UK. The in vitro Toxicology Assay Kit Lactate Dehydrogenase Based, thiazolyl blue tetrazolium bromide (MTT), Dulbecco’s modified Eagle’s medium (DMEM), DMEM/Nutrient Mixture F-12 Ham (DMEM/F12 Ham), fetal bovine serum (FBS), penicillin and streptomycin were obtained from Sigma Aldrich (St. Louis, MO, USA). Cell Proliferation ELISA BrdU (bromodeoxyuridine) was obtained from Roche Diagnostics GmbH (Penzberg, Germany). Eagle’s Minimum Essential Medium (EMEM) was obtained from the American Type Culture Collection (ATCC, Manassas, VA, USA). Redistilled phenol, Bradford reagent, 2,2′-azinobis-(3-ethylbenzothiazoline-6-sulfonic acid) (ABTS^•+^), bovine serum albumin (BSA), and gallic acid were purchased from Sigma-Aldrich Fine Chemicals (St. Louis, MO, USA). Soybean 15-lipooxygenase, xanthine oxidase, catalase, Trolox, 2,2′-azobis (2-methylpropionamide) dihydrochloride (AAPH), and linoleic acid were provided by Sigma-Aldrich Chemical Co. (St. Louis, MO, USA). Fluorescein sodium salt was purchased from Roth (Karlsruhe, Germany). D_2_O was obtained from Deutero GmbH (Kastellaun, Germany).

All other chemicals and solvents were of analytical grade and were purchased from Avantor Performance Materials Poland (Gliwice, Poland).

Colorimetric and fluorescence measurements were performed on a 96-well transparent and black microplates (both from Nunclon, Nunc; Roskilde, Denmark), respectively, using an Infinite Pro 200F microplate reader from Tecan Group Ltd. (Männedorf, Switzerland). The evaporation of extracts was conducted using a Heidolph Basis Hei-VAP Value evaporator (Schwabach, Germany). Lyophilization was performed in the Free Zone 1 apparatus (Labcono, Kansas City, KS, USA).

### 2.3. Extraction of Crude Polysaccharides (CPS)

**Figure 1:**
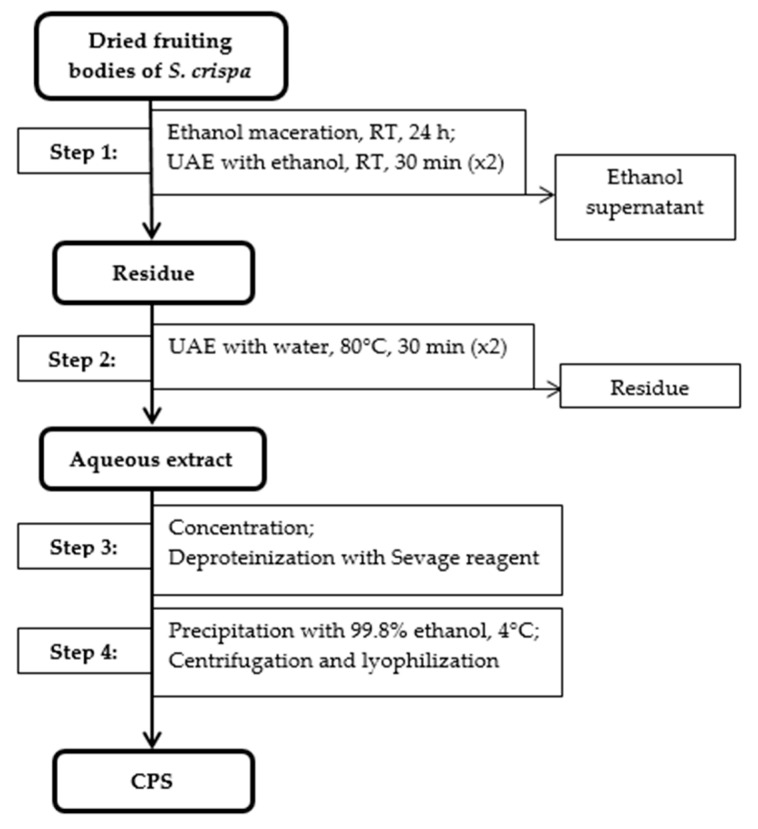
Schematic diagram of extraction of crude polysaccharides from fruiting bodies of *S. crispa* (CPS). Abbreviations: RT—room temperature; UAE—ultrasonic-assisted extraction.

Freeze-dried and milled fruiting bodies of *S. crispa* (10 g) were macerated with 99.8% ethanol (100 mL) for 24 h at room temperature and then extracted two times with 99.8% ethanol using ultrasonic-assisted extraction (UAE) to remove low molecular weight compounds. Then, the supernatant was removed, and the residue was extracted two times for 30 min with distilled water (200 mL) by UAE at 80 °C. The combined aqueous extracts were concentrated under vacuum to 20 mL. The concentrated extracts were further purified by deproteinization using the Sevage reagent (chloroform/isoamyl alcohol, 4:1, *v*/*v*) [20]. Subsequently, polysaccharides were precipitated with cold 99.8% ethanol (1:4, *v*/*v*) and kept overnight in the refrigerator at 4 °C. The resulting precipitates of crude polysaccharides were collected by centrifugation (9055 G, 15 min) and lyophilized (Figure 1). The polysaccharide yields (%) were calculated as described below:

Yield (%, *w*/*w*) = Weight of extracted polysaccharides/Weight of dried material × 100 (1)

### 2.4. Chemical Composition of Crude Polysaccharides

#### 2.4.1. Sugar Content Determination

The total sugar content was determined with the phenol–sulphuric acid method [21] using glucose as a standard. One microliter of the sample was mixed with 25 µL of an 80% (*w*/*v*) aqueous phenol solution in a test-tube and 2.5 mL of concentrated H_2_SO_4_ was added. The absorbance was measured at λ = 485 nm. The results were converted into mg of glucose equivalents and expressed as % of CPS.

#### 2.4.2. Protein Content Determination

The protein concentration in CPS was determined with the Bradford method as previously described [22] using bovine serum albumin as a standard. The reaction mixture consisted of 1 mL of Bradford Reagent and 0.1 mL of the sample. Absorbance at λ = 595 nm was measured. The results were expressed as % of CPS.

#### 2.4.3. Total Phenolic Content Determination

The content of phenolic compounds was assayed using the method described in detail by [23] using gallic acid as a standard. Twenty microliters of the sample was added to 20 µL of the diluted Folin–Ciocalteu reagent (with water 1:4, *v*/*v*) followed by the addition of 160 µL of sodium carbonate (75 g/L). The absorbance was measured at λ = 680 nm after 20 min of incubation. The results were expressed as % of CPS.

### 2.5. Structural Analysis

#### 2.5.1. Sugar Composition

The sugar composition of CPS was determined using sugar analysis. The polysaccharide fraction (~0.5 mg) was hydrolyzed with trifluoroacetic acid (2 M TFA, 2 h at 120 °C), reduced with sodium borohydride, and acetylated with acetic anhydride in the presence of sodium acetate (120 °C for 2 h). The alditol acetate derivatives obtained were analyzed by GC–MS using a Shimadzu GC–MS QP2010SE system equipped with a Rtx-5 (30 m) capillary column [24].

#### 2.5.2. NMR Spectroscopy

CPS (~5 mg) was dissolved in 1 mL of 99% D_2_O to replace all exchangeable protons, freeze-dried, and dissolved in 0.75 mL of 99.9% D_2_O for measurements. The ^1^H NMR spectrum was recorded at 40 °C using a Bruker Avance III 500 MHz spectrometer. ^1^H chemical shifts were referenced to acetone (δH 2.225).

### 2.6. α- and β-Glucan Determination

The content of glucans in *S. crispa* was determined using the Megazyme Mushroom and Yeast Beta-Glucan Assay Kit according to the manufacturer’s instructions. To determine the total glucan content, milled *S. crispa* was hydrolyzed with concentrated hydrochloric acid and neutralized with 2 M potassium hydroxide. Then, the digestion with exo-1,3-β-glucanase (20 U/mL) plus β-glucosidase (4 U/mL) in 200 mM sodium acetate buffer (pH 5.0) was performed. To measure the glucan content, glucose oxidase/peroxidase and 4-aminoantipyrine in p-hydroxybenzoic acid and sodium azide (GOPOD) reagent was added. The absorbance was measured at λ = 510 nm against the GOPOD reagent blank. For measuring the content of α-glucans, a milled mushroom sample was hydrolyzed in 2 M KOH and neutralized with 1.2 M sodium acetate buffer (pH 3.8). Then, amyloglucosidase (1630 U/mL) and invertase (500 U/mL) were added into the hydrolysate and incubated at 40 °C for 30 min. After enzymatic hydrolysis, the GOPOD reagent was added and incubated at 40 °C for 20 min. The absorbance was measured at λ = 510 nm against the reagent blank including the sodium acetate buffer instead the sample tested. The total glucan and α-glucan contents were calculated by comparison to the D-glucose standard. The β-glucan content was determined by subtracting the α-glucan from the total glucan content. All measurements were taken a minimum of three times. The results were expressed as g/100 g of dry weight and are expressed as the mean ± standard deviation (SD).

### 2.7. Anticancer Potential—In Vitro Studies

#### 2.7.1. Cell Lines

The human colon epithelial cell line CCD 841 CoN and the human colon adenocarcinoma cell line Caco-2 were purchased from the American Type Culture Collection (ATCC, Manassas, VA, USA). The human colon adenocarcinoma cell lines HT-29 and LS180 were obtained from the European Collection of Cell Cultures (ECACC, Centre for Applied Microbiology and Research, Salisbury, UK). The CCD 841 CoN cells were grown in DMEM. The Caco-2 cells were grown in EMEM. The HT-29, and LS180 cells were grown in DMEM/F12 Ham. All media were supplemented with penicillin (100 U/mL), streptomycin (100 mg/mL), and FBS in the amount 10% (CCD841 CoN, LS180, HT-29) or 20% FBS (Caco-2). The cells were maintained in a humidified atmosphere of 95% air and 5% CO_2_ at 37 °C.

#### 2.7.2. MTT Assay

The cells were seeded on 96-well microplates at a density of 3 × 10^4^ cells/mL (cancer cells) and 5 × 10^4^ cells/mL (normal cells). On the following day, the culture medium was removed, and the cells were exposed to serial dilutions (10, 25, 50, and 100 μg/mL) of the crude polysaccharide extract from *S. crispa* prepared in the fresh medium with the standard content of FBS. The cell metabolic activity was assessed after 96 h of incubation in standard conditions (5% CO_2_, 37 °C) by means of the MTT assay, in which the yellow tetrazolium salt (MTT) was metabolized by viable cells to purple formazan crystals. After the incubation period, MTT solution (5 mg/mL in PBS) was added into the cells for 3 h. The resultant crystals were solubilized overnight in SDS buffer at pH 7.4 (10% SDS in 0.01 M HCl), and the product was quantified spectrophotometrically by measuring the absorbance at a wavelength of λ = 570 nm using the microplate reader (BioTek ELx800, Highland Park, Winooski, VT, USA). The results were presented as a percentage of metabolic activity of cells treated with the investigated compound versus cells grown in the control medium (indicated as 100%).

#### 2.7.3. BrdU Assay

Cells were seeded on 96-well microplates at a density of 3 × 10^4^ cells/mL (cancer cells) and 5 × 10^4^ cells/mL (normal cells). On the following day, the culture medium was removed, and the cells were exposed to serial dilutions (10, 25, 50, and 100 μg/mL) of the crude polysaccharide extract from *S. crispa* prepared in the fresh medium with a standard content of FBS. After 48 h of incubation in standard conditions (5% CO_2_, 37 °C), the impact of CPS on DNA synthesis was measured by a colorimetric immunoassay, namely the Cell Proliferation ELISA BrdU according to the manufacturer’s instructions. The test measures cell proliferation by quantitating BrdU (analog of thymidine, 5-bromo-20-deoxyuridine) incorporated into the newly synthesized DNA in proliferating cells. Absorbance was measured at a λ = 450 nm wavelength using the microplate reader (BioTek ELx800, Highland Park, Winooski, VT, USA). The results were presented as a percentage of the BrdU incorporation to DNA in cells treated with the investigated compound versus the cells grown in the control medium (indicated as 100%).

#### 2.7.4. LDH Assay

Cells were seeded on 96-well microplates at a density of 5 × 10^4^ cells/mL (cancer cells) and 1 × 10^5^ cells/mL (normal cells). On the following day, the culture medium was removed, and the cells were exposed to serial dilutions (10, 25, 50, and 100 μg/mL) of the crude polysaccharide extract from *S. crispa* prepared in the fresh medium supplemented with 2% FBS. After 24 h of incubation in standard conditions (5% CO_2_, 37 °C) the culture supernatants were collected in new 96-well microplates, which were used to perform the lactate dehydrogenase (LDH) assay following the manufacturer’s instruction. The test was based on the measurement of lactate dehydrogenase (LDH) released into the culture medium upon damage to the cell plasma membrane. The absorbance was recorded on a microplate reader (BioTek ELx800, Highland Park, Winooski, VT, USA) at a wavelength of λ = 450 nm. The results were presented as the percentage of LDH released from cells treated with the tested compound versus the cells grown in the control medium (indicated as 100%).

### 2.8. Anti-Inflammatory Activity

#### 2.8.1. Inhibition of COX Activity

The ability of crude polysaccharides from *S. crispa* to inhibit cyclooxygenase activity was determined in vitro with the use of the COX (ovine) Colorimetric Inhibitor Screening Assay Kit. Briefly, 10 µL of the polysaccharide sample (1 mg/mL, in 5% DMSO) were added to the reaction mixture containing 150 μL of assay buffer, 10 μL of heme, and 10 μL of the enzyme (either COX-1 or COX-2). Peroxidase activity can be assayed colorimetrically by monitoring the appearance of oxidized *N*,*N*,*N*’,*N*’-tetramethyl-*p*-phenylenediamine (TMPD) at λ = 590 nm. Acetylsalicylic acid (1 mM) was used as a control. The percent COX inhibition was calculated as shown below:

COX inhibition activity (%) = 1 − Absorbance of the inhibitor well at λ = 590 nm/Absorbance of the 100% initial activity without the inhibitor at λ = 590 nm × 100

#### 2.8.2. Inhibition of LOX Activity

Inhibition of 15-lipoxygenase (LOX) was determined as previously described with some modifications using soybean 15-LO, generally regarded as predictive for the inhibition of the mammalian enzyme [25]. The LOX inhibition was determined spectrophotometrically at 20 °C by measuring the increase in absorbance at λ = 234 nm over a 2 min period. The reaction mixture consisted of 0.2 M borate buffer (pH 9.00), the CPS sample (1 mg/mL, in water), the enzyme (167 U/mL), and linoleic acid (134 µM) as a substrate. Acetylsalicylic acid (1 mM) was used as a control. All measurements were carried out in triplicate. The LOX inhibitory activity was expressed as the percentage inhibition of LOX in the above assay mixture system in relation to the control without the inhibitor (indicated as 100%). The mode of inhibition on the enzyme was shown using the Lineweaver–Burk plot.

### 2.9. Antioxidant Activity

#### 2.9.1. Antiradical Activity against ABTS^•+^

Antiradical activity against ABTS^•+^ was determined with the method described by [26]. Twenty microliters of the sample was mixed with 180 µL of the ABTS^•+^ solution (0.096 mg/mL). The mixture was shaken and incubated for 6 min. The absorbance was measured at λ = 734 nm. The scavenging activity of the extracts was determined using the following formula:

% Reduction = [(AB − AA)/AB] × 100(2)

where AB is the absorption of the control sample (ABTS^•+^ solution and solvent instead of the sample), and AA is the absorption of the sample with ABTS^•+^ reagent. Six dilutions of CPS were examined to plot a dose–response curve and determine the EC_50_ value (concentration of CPS providing 50% of activity).

#### 2.9.2. Reducing Power

Reducing power (RP) was determined using the method described by [27], in which 2.5 mL of the sample was mixed with 2.5 mL of phosphate buffer (200 mM, pH 6.6) and 2.5 mL of 1% aqueous solution of K_3_[Fe(CN_6_)]. After 20 min of incubation at 50 °C, 0.5 mL of 10% trichloroacetic acid was added. The mixture was centrifuged at 25× *g* for 10 min. 2.5 mL pf the upper layer of solution was mixed with 2.5 mL of distilled water and 0.5 mL of FeCl_3_. The absorbance was measured at λ = 700 nm. Six dilutions of CPS were examined to plot a dose–response curve. The result was expressed as the concentration of polysaccharides providing 50% of activity based on a dose-dependent mode of action (EC_50_).

#### 2.9.3. Inhibition of Lipid Peroxidation

The degree of inhibition of linoleic acid peroxidation (LPO) was performed according to [28] described in detail by [29]. Absorbances of reaction mixtures were measured at λ = 480 nm. Six dilution of CPS were examined to plot a dose–response curve. The result was expressed as the concentration of polysaccharides providing 50% of activity based on a dose-dependent mode of action (EC_50_).

#### 2.9.4. Metal Chelating Activity

Chelating power (CHP) was determined with the method described by [30]. The reaction mixture consisted of 5 mL of CPS, 0.1 mL of FeCl_2_ (2mM) and 0.2 mL of ferrozine (5 mM). Absorbance was measured at λ = 562 nm after 10 min of incubation at room temperature. Six dilutions of CPS were examined to plot a dose–response curve. The result was expressed as the concentration of polysaccharides providing 50% of activity based on the dose-dependent mode of action (EC_50_).

#### 2.9.5. Oxygen Radical Absorbance Capacity (ORAC) Assay

The analysis was performed according to the slightly modified method proposed by [31] and described in detail by [26]. The sample activity was expressed as µM of Trolox/mg of crude polysaccharides. All determinations were carried out in triplicate.

#### 2.9.6. Catalase Activity Assay

The influence of *S. crispa* crude polysaccharides on catalase (CAT) activity was assayed with the method developed by [32] with some modification. The assay mixture consisted of phosphate buffer (0.05 M, pH 7.0), the CPS sample (1 mg/mL, in water), the enzyme solution (60 U/mL), and H_2_O_2_ (0.019 M). The decomposition of H_2_O_2_ was determined directly by the extinction at λ = 240 nm per unit time (3 min) was used as a measure of catalase activity.

#### 2.9.7. Inhibition of Xanthine Oxidase Activity

Inhibition of xantine oxidase (XO) was determined as previously described with some modifications [33]. The assay mixture consisted of phosphate buffer (1/15 M, pH 7.5), the CPS sample (1 mg/mL, in water), the enzyme solution, and xanthine as a substrate. The assay mixture was incubated at 30 °C with the absorbance (λ = 295 nm) measured spectrophotometrically over a 2 min period. The XO inhibitory activity was expressed as the percentage inhibition of XO in the assay mixture system.

### 2.10. Statistical Analysis

All results were expressed as the mean ± standard deviation (SD) from three replications. Calculations were performed in STATISTICA 10.0 (StatSoft Poland, Cracow, Poland). The data from the anticancer activity determination were presented as the mean value and standard error of the mean (SEM). Statistical analysis was performed using one way-ANOVA with the Tukey post hoc test and column statistics used for comparisons. Significance was accepted at *p* < 0.05. The IC_50_ value (concentration causing proliferation inhibition by 50% compared to the control) was calculated according to the Litchfield and Wilcoxon method [34].

## 3. Results

### 3.1. Chemical Composition of S. crispa Crude Polysaccharides and Contents of α- and β-Glucans

Crude polysaccharides (CPS) were precipitated with cold ethanol from the aqueous extract of *S. crispa* with an efficiency of 9.5% of d.w. In the first stage, the chemical composition of CPS was investigated by total sugar, protein, and total phenolic content analysis. The results (Table 1) indicate that CPS consists mainly of sugars (60.5%). Proteins and phenolics are only present in trace amounts.

Sugar analysis of CPS revealed the presence of three different hexoses and one 6-deoxyhexose in the molar ratio ~1.0:0.4:0.2:0.1. Monosaccharides were identified as glucose, galactose, mannose, and fucose, respectively, by the comparison of the retention times with the authentic standards.

**Figure 2:**
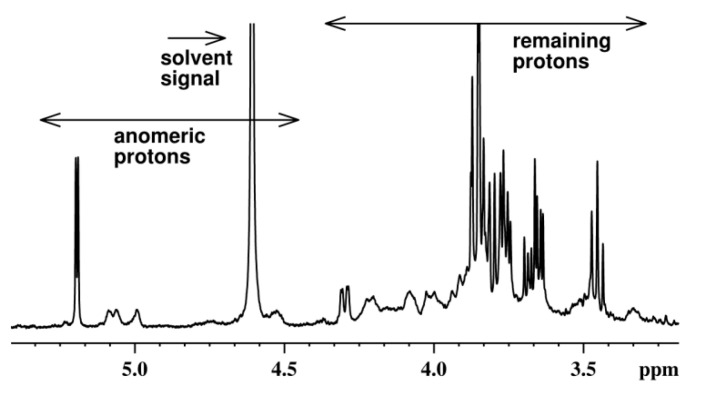
^1^H NMR spectrum of the polysaccharide fraction isolated from *S. crispa.*

The profile of the ^1^H NMR spectrum (Figure 2) is characteristic of polysaccharides. It contains some anomeric proton signals in the region of 4.5–5.3 ppm and the remaining proton signals in the region of 3.0–4.3 ppm.

The amounts of total as well as α- and β-glucans in *S. crispa* were determined with the enzymatic method. The results are presented in Table 2. The total content of glucans in the cauliflower mushroom was 29.96 g/100 g of dry weight. It was found that the amount of β-glucans was higher than that of α-glucans. β-glucans accounted for 91.86% of the total glucans in *S. crispa*.

### 3.2. Biological Activity of CPS

#### 3.2.1. Anticancer Potential—In Vitro Studies

**Figure 3:**
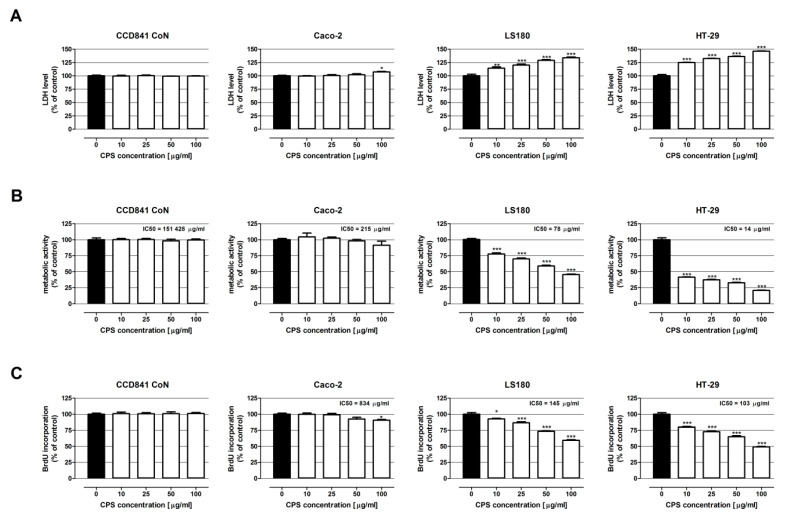
Antiproliferative and cytotoxic effect of crude polysaccharides isolated from *S. crispa* on human colon epithelial cell line CCD841 CoN and human colon adenocarcinoma cell lines: Caco-2, LS180, and HT-29. The cells were exposed to the culture medium alone (control) or the crude polysaccharides from *S. crispa* (CPS) at concentrations of 10, 25, 50, and 100 µg/mL for 24 h (LDH assay), for 48 h (BrdU assay) and for 96 h (MTT assay). CSP cytotoxicity was measured photometrically by means of the LDH assay (**A**), while the antiproliferative impact of CSP was examined photometrically by means of the MTT assay (**B**) and BrdU assay (**C**). Results are presented as mean ± SEM of at least 4 measurements. * *p* < 0.05 versus control, ** *p* < 0.01 versus control, *** *p* < 0.001 versus control, one-way ANOVA test; post-test: Tukey.

The crude polysaccharides from the cauliflower mushroom were subjected to both antiproliferative activity determination (MTT and BrdU assay) and cytotoxicity examination (LDH assay) using human colon epithelial cell line CCD841 CoN and three human colon adenocarcinoma cell lines: Caco-2, LS180, and HT-29. The results are presented in Figure 3.

The results of the LDH assay have shown that the crude polysaccharides isolated from *S. crispa* were not cytotoxic to the human colon epithelial CCD841 CoN cells. On the contrary, CPS damaged the cell membranes of all investigated colon cancer cell lines. The most significant changes were observed in the HT-29 cells, wherein the LDH level in response to CPS increased from 124.7% (10 µg/mL) to 146.1% (100 µg/mL). A slightly weaker cytotoxic effect was induced by the tested polysaccharides in the LS180 cells; 10 µg/mL CPS increased LDH release by 14.3%, while 100 µg/mL CPS accelerated the cell membrane damage by 33.8%. In the case of the Caco-2 cells, only the highest concentration of *S. crispa* polysaccharides elevated the LDH level by 7.2%.

The MTT test demonstrated no impact of the CPS on the metabolic activity of both CCD841 CoN and Caco-2 cells. At the same time, the polysaccharides exerted a significant antiproliferative effect on the LS180 and HT-29 cells (IC50 LS180 = 78 µg/mL; IC50 HT-29 = 14 µg/mL). The strongest inhibition of proliferation was observed in the HT-29 cells, wherein the lowest and the highest investigated concentrations of CPS caused a decrease in cancer cell proliferation by 58.6% and 79.1%, respectively. The LS180 cells were less sensitive to the CPS; nevertheless, even the lowest dose of *S. crispa* polysaccharides reduced the cancer cell proliferation by 22.6%.

The BrdU assay (more sensitive and specific antiproliferative assay) revealed no influence of CPS on DNA synthesis in human colon epithelial CCD841 CoN cells. Simultaneously, *S. crispa* polysaccharides decreased the proliferation of all investigated human colon cancer cells in a dose-dependent manner (IC50 Caco-2 = 834 µg/mL; IC50 LS180 = 145 µg/mL; IC50 HT-29 = 103 µg/mL). Among the examined colon cancer cell lines, Caco-2 was the most resistant to the antiproliferative abilities of CPS. The significant inhibition of DNA synthesis (9.4%) was noted only after the treatment with polysaccharides at a concentration of 100 µg/mL. On the contrary, the strongest anticancer effect was observed in HT-29 cells, wherein even 10 μg/mL of CPS inhibited the cell proliferation by 20.1%, while 100 μg/mL of CPS caused a 50.8% reduction in DNA synthesis. LS180 cells were more resistant to CPS treatment than HT-29, and the decrease in BrdU incorporation was in the range from 7.3% (10 μg/mL) to 40.6% (100 μg/mL).

#### 3.2.2. Antioxidant Activity

We determined the antioxidant activity of CPS using different methods involving different modes of antioxidant action. The results are presented in Table 3. They revealed that the crude polysaccharide from *S. crispa* exhibited moderate potential in antiradical activity against ABTS^•+^ with the EC_50_ level of 16.27 mg/mL CPS. CPS was found to have the highest antioxidant potential in the determination of reducing properties (EC_50_ = 0.82 mg/mL) and chelating activity (EC_50_ = 0.76 mg/mL). Our study also included an evaluation of the ability of CPS to inhibit lipid peroxidation. The EC_50_ value obtained indicates a high activity of CPS (2.76 mg/mL). The antioxidant activity of CPS was then evaluated with the ORAC assay, giving the result 168.51 ± 0.21 µM Trolox/g CPS.

The antioxidant activity of CPS was also evaluated using enzymatic assays with xanthine oxidase (XO) and catalase (CAT). The results are presented in Table 4. CPS was found to have no influence on XO activity, as neither the activation nor inhibition of this enzyme was observed. The catalase activity assay revealed that CPS did not activate this enzyme, which suggests an absence of antioxidant activity. On the contrary, our study revealed that catalase activity was inhibited by CPS in 32.74% at the CPS concentration of 5 mg/mL.

#### 3.2.3. Anti-Inflammatory Activity

**Figure 4:**
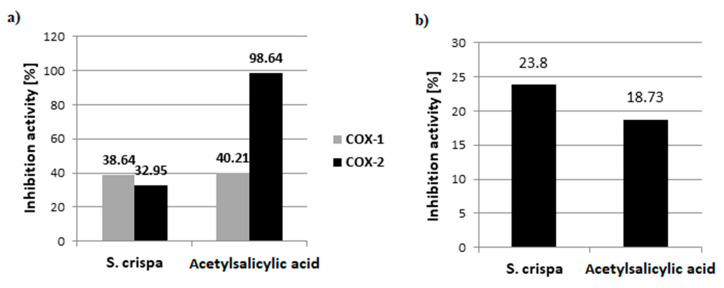
Inhibition of: (**a**) cyclooxygenase-1, cyclooxygenase-2 and (**b**) lipooxygenase activity by crude polysaccharides from *S. crispa* (5 mg/mL) expressed in %. Acetylsalicylic acid (1 mM) was used as a control.

In our study, the crude polysaccharides from *S. crispa* were tested for their ability to inhibit the activity of cyclooxygenase and lipooxygenase. The results are presented in Figure 4. CPS was found to inhibit COX-1 activity at a level similar to that of acetylsalicylic acid, i.e., a commonly known nonsteroidal anti-inflammatory drug. In the case of COX-2, the CPS activity, estimated at 32.95%, was lower than that of acetylsalicylic acid. The LOX enzyme, which is involved in the development of inflammation in the human organism, was inhibited by 23.8%. This result was higher than that obtained for acetylsalicylic acid (18.73%).

**Figure 5:**
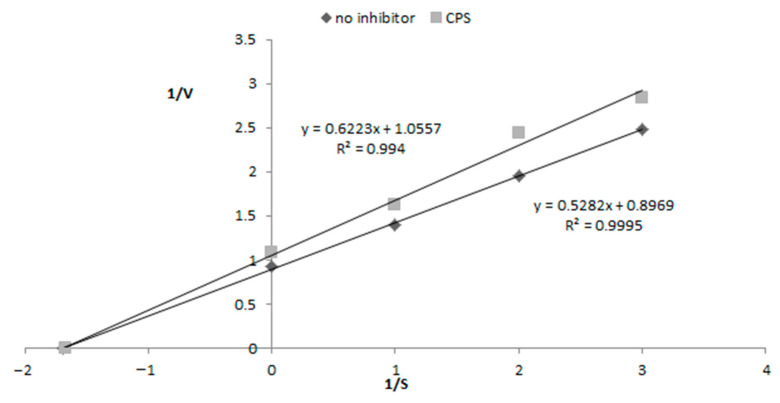
Mode of 15-lipoxygenase (LOX) inhibition by CPS determined by the Lineweaver–Burk plot.

The mode of LOX inhibition by CPS was determined using the Lineweaver–Burk plot presented in Figure 5. The y-intercept and the slope of a Lineweaver–Burk plot indicate that CPS caused the noncompetitive inhibition of LOX, in which the inhibitor binds to an allosteric site, resulting in the decreased efficacy of the enzyme. In this mode of inhibition, CPS shares the same affinity for both the enzyme and the enzyme–substrate complex and the enzyme is prevented from forming its product [35]. 

## 4. Discussion

### 4.1. Chemical Composition of CPS

*S. crispa* is a medicinal mushroom with a long history of use in traditional Asian therapies. The fruiting body of *S. crispa* contains approximately 13.4 g of protein, 21.5 g of carbohydrates, and 2.0 g of fat per 100 g of dry weight [19]. Ultrasonic-assisted extraction (with water as an eluent) was used to obtain polysaccharides, since the ultrasonic enhancement of the process causes the disruption of cell walls, reduces particle size, and enhances transfer of cell components resulting in better extraction efficiency than traditional techniques [36]. The isolation of the crude polysaccharides was preceded by preliminary extraction with ethanol (to remove small molecules) and involved the deproteinization method (with Sevage reagent). Therefore, proteins and phenolics were finally present in CPS only in trace amounts. Sugars constituted the main group of compounds in crude polysaccharides (Table 1). They were identified as glucose, galactose, mannose, and fucose with the GC–MS method. Structural analysis based on the ^1^H NMR spectrum of CPS confirmed that it contained polysaccharides (Figure 2). The ^1^H NMR spectrum contains some anomeric proton signals in the region of 4.5–5.3 ppm and the remaining proton signals in the region of 3.0–4.3 ppm. The signals in the region of 4.5–4.6 ppm are characteristic for β-monosaccharides and confirmed the presence of β-glucan, which was determined with the enzymatic method and described in Table 2.

According to our knowledge, previous structural studies of polysaccharides isolated from *S. crispa* revealed the presence of a glucan, namely of a β-(1-3)-d-glucan backbone with a single β-(1-6)-d-glucosyl side branching units at every three residues [37].

Various medicinal properties of *S. crispa* are mostly attributed to the presence of β-glucans. According to Japan Food Research Laboratories (Tokyo, Japan), the β-glucan content in *S. crispa* is more than 40% of the dry weight [38]. Our results revealed a lower amount of β-glucans (Table 2). This discrepancy may result from the origin of the mushrooms, since the *S. crispa* used in this study was collected from the natural environment in Poland, while Asian species are mostly cultivated. Moreover, the taxonomy and systematics of *Sparassis* Fr. species have been refined according to phylogenetic relationships and placement. It was proposed that *Sparassis* should be classified into three groups: *S. crispa* from Europe and eastern North America, *S. radicata* from western North America, and *S. latifolia* from Asia [39,40]. There are no available data showing possible differences in the chemical composition of each species from the different regions.

### 4.2. Biological Activity of CPS

The antitumor activity of *S. crispa* β-glucan has been previously examined alone or in combination with some chemotherapeutics. As demonstrated by [41], polysaccharide fractions from *S. crispa* exhibited anticancer activity to the solid form of Sarcoma 180 in mice and showed a hematopoietic response to cyclophosphamide-induced leukopenia in mice. Moreover, β-glucan from *S. crispa* suppressed the number and growth of lung metastatic colonies in B16-BL6-bearing mice [42].

To the best of our knowledge, there is no information about the chemopreventive properties of polysaccharide fractions from *S. crispa* against colon cancer. Therefore, crude polysaccharides from the wild growing cauliflower mushroom were subjected to both antiproliferative activity determination and cytotoxicity examination. The studies were performed on human colon epithelial cell line CCD841 CoN and on three different human colon adenocarcinoma cell lines, which represent the successive stages of colon cancer development according to Dukes classification (Caco-2: Dukes B, LS180: Dukes B, HT-29: Dukes C).

The present study revealed in vitro the great bioactive properties of crude polysaccharides isolated from the wild growing cauliflower mushroom. They were found to be non-toxic to normal human colon epithelial cells; simultaneously, they significantly inhibited the proliferation of the human colon cancer cells and destroyed their cell membrane integrity. It needs to be highlighted that CPS had the lowest efficiency in the elimination of the Caco-2 cancer cells, which are the most differentiated but the least invasive cell line among the investigated ones. On the contrary, the HT-29 cells representing the advanced stage of colon cancer development were the most sensitive to the anticancer effect of CPS [43,44]. There was a positive correlation between the CPS anticancer activity and the colon cancer invasiveness and undifferentiation. The *S. crispa* polysaccharides had better activity in the more destruction-resistant colon cancer cells. Obviously, this observation is worth further verification in more advanced studies, e.g., in in vivo models.

Inflammation is a host response to infections or tissue injury that occurs throughout a complex set of interactions among soluble factors and cells. In normal conditions, the inflammatory response is self-limiting. However, prolonged inflammation can cause many diseases [45]. There was a strong relationship between long-term inflammation and the development of colon cancer. Colitis-associated cancer (CAC) is a subtype of colorectal cancer linked to inflammatory bowel disease (IBD). Chronic inflammation in CAC is responsible for oxidative damage to DNA, resulting in p53 mutations observed in tumor cells and the inflamed but nondysplastic epithelium [46]. Therefore, searching for natural anti-inflammatory agents without or with low toxic effects seems necessary. There are numerous studies indicating that mushroom polysaccharides, including β-glucans, possess immunomodulatory or anti-inflammatory activities [47]. Inducible cyclooxygenase-2 (COX-2) and 5-lipooxygenase (5-LOX) are among the best known inflammatory biomarkers produced in the human body. Previous research revealed that a non-aqueous fraction from *S. crispa* extract inhibited the production of PGE_2_ via the downregulation of the expression of COX-2 [48]. A study conducted by [49] revealed that the water extract of *S. crispa* suppressed mast cell-mediated allergic inflammation by regulating calcium, MAPK, and NF-κB. Moreover, several phthalides from cauliflower mushroom exerted an inhibitory effect on LPS-stimulated NO and PGE_2_ production by RAW264 cells [50]. It was demonstrated that a branched β-glucan from *S. crispa* induced macrophages to produce several mediators, including the inflammatory cytokines interleukin-1 (IL-1), IL-6, tumor necrosis factor-a (TNF-a), and nitric oxide (NO) [51]. Our study demonstrates the anti-inflammatory potential of CPS in the direct inhibition of pro-inflammatory enzymes such as COX-2 and LOX. Further studies are necessary to establish whether there are any additional mechanisms of the anti-inflammatory activity of *S. crispa* polysaccharides.

The overproduction of reactive oxygen species (ROS) in the human body leads to oxidative stress, causing numerous pathological conditions, including development of colon cancer. It occurs through high susceptibility to the influence of pro-oxidative and toxic factors and the following increase in the proliferation of cancer cells [52]. Therefore, it seems reasonable to introduce antioxidants to everyday diet as chemopreventive agents. Mushroom polysaccharides from edible species are interesting candidates for this purpose. The present study has revealed that the crude polysaccharides from *S. crispa* possess moderate antiradical activity and strong reducing properties and chelating activity as well as high potential in inhibition of lipid peroxidation. It has already been found that the mechanism of the antioxidant activity of polysaccharides from mushrooms depends on their chemical structure. Molecular weight, monosaccharide composition, chain conformation, and structural configuration may affect the antioxidant capacity of this group of compounds [53]. In the case of free radical scavenging, polysaccharides are believed to have greater potential than monosaccharides. This activity may be determined by the size of molecules and the type of binding in the side branches of the main chain of the polysaccharides; nevertheless, their antioxidative activity is still rather moderate [36]. A previous study reported the antioxidant activity of polysaccharides from *S. crispa* revealed in the ABTS^•+^ assay. The authors found that the scavenging effect of two polysaccharides at the concentration of 5.0 mg/mL reached 84.02 and 80.70%, while the activity of the standard compound (vitamin C) was 97.48% [53]. Researchers demonstrated the antioxidant activity of crude polysaccharides from *Cordyceps miltaris*, i.e., one of the most famous functional and medicinal mushrooms, using methods similar to those employed in our study. The reducing power (expressed as the EC_50_ value) of various polysaccharides from *C. militaris* ranged from 1.06 to 6.07 mg/mL, and the chelating activity ranged from 3.09 to 7.74 mg/mL [54]. In comparison with our study, this implies that the polysaccharide fraction from *S. crispa* has significantly higher antioxidant properties (Table 3) than the polysaccharides from *C. militaris*. CPS was found to be able to inhibit lipid peroxidation on the hemoglobin-catalyzed the peroxidation of linoleic acid. Moreover, the ORAC assay, which relies on the utilization of the AAPH-derived peroxyl radical imitating lipid peroxyl radicals, was involved in the lipid peroxidation chain reaction in vivo [55], confirmed the antioxidant properties of polysaccharides from the cauliflower mushroom. The antioxidant activity of crude natural polysaccharides may be attributed to the presence of various compounds in mushroom extracts, e.g., phenolic compounds [56]. However, our current research shows that crude polysaccharides also exhibit moderate or even high antioxidant potential, which is not related to the total phenolic content. Antioxidant properties may be related especially to the presence of β-glucans in the polysaccharide fraction from mushrooms [54]. Certainly, further studies of the function–structure relationship are necessary.

Overproduction of ROS in vivo can also occur through some enzyme-mediated reactions. Xanthine oxidase constitutes one of the main enzymatic sources of ROS in vivo due to its participation in the oxidative damage resulting from the reperfusion of ischemic tissues, brain edema and injury, or vascular permeability changes [57]. The inhibition of XO by CPS was studied in our study. However, the results indicate no influence of CPS on the XO activity.

We also used another enzymatic method for testing antioxidant activity, i.e., the catalase assay. Catalase protects cells from oxidative stress through the decomposition of hydrogen peroxide to water and oxygen [32]. Therefore, the promotion of catalase activity is one of the indirect mechanisms of antioxidant activity that is beneficial from the point of view of normal cell physiology. On the other hand, it was shown that catalase contributed to the increased resistance of cancer cells to pro-oxidant drugs (especially in H_2_O_2_-mediated processes). The inhibition of catalase expression and activity results in increased oxidative stress in cancerous cells. This provides new insight into understanding the possible anticancer properties of some compounds [58,59]. Therefore, searching for catalase inhibitors seems reasonable in order to design synergistic agents for anti-cancer drugs, which may help to sensitize drug-resistant cancer cells [60]. Our study revealed that the *S. crispa* polysaccharides inhibited catalase activity. Therefore, their ability seems to be suitable to be applied for the enhancement of anti-cancer chemotherapy.

## 5. Conclusions

*S. crispa* is a popular edible and medicinal mushroom and a rich source of polysaccharides, including β-glucans. Our in vitro study revealed the promising chemopreventive potential of its crude polysaccharide (CPS) based on several different mechanisms of action. CPS was found to inhibit the proliferation of colon cancer cells significantly without a concurrent harmful effect on normal cells. Moreover, antioxidant and anti-inflammatory activities fitting into the strategy of colon cancer prevention were proved. Since colon cancer was found to be related to food and lifestyle, searching for natural chemopreventive agents administered as part of the daily diet seems crucial. Polysaccharides from *S. crispa* can be a valuable addition to human diet as nutraceuticals or functional food ingredients. The fruiting bodies of cauliflower mushroom may be consumed as a part of habitual regular diet. Moreover, CPS might be used as bioactive additives incorporated in various food products accessible for consumers. The potential use of polysaccharides from *S. crispa* in supporting colon cancer prevention and treatment in relation to their chemical structure will be addressed in further research.

## Figures and Tables

**Table 1:** Chemical composition of *S. crispa* crude polysaccharides (CPS). Abbreviations: TPC–total phenolic content.

Sugar Content (% of CPS)	Protein Content (% of CPS)	TPC (% of CPS)
60.5 ± 0.98	0.48 ± 0.01	0.15 ± 0.00

**Table 2:** Total glucan, α-glucan, and β-glucan content in *S. crispa* fruiting bodies expressed in g per 100 g of dry weight.

Total Glucan (g/100 g d.w.)	α-Glucan (g/100 g d.w.)	β-Glucan (g/100 g d.w.)
29.96 ± 0.59	2.44 ± 0.03	27.52 ± 0.32

**Table 3:** Antioxidant activity of crude polysaccharides from *S. crispa* determined with different methods expressed as EC_50_ values (mg/mL). The results are presented as the mean ± standard deviation of 3 measurements. Abbreviations: ABTS—antiradical activity against ABTS^•+^, RP—reducing power, LPO—inhibition of lipid peroxidation, CHP—metal chelating activity, EC_50_—the polysaccharides concentration providing 50% of activity based on dose-dependent mode of action.

Antioxidant Assay	CPS Antioxidant Activity EC_50_ ± SD [mg/mL]
ABTS^•+^	16.27 ± 3.42
RP	0.82 ± 0.01
LPO	2.76 ± 0.02
CHP	0.76 ± 0.19

**Table 4:** Antioxidant activity of crude polysaccharides from *S. crispa* determined by enzymatic assays. Abbreviations: XO—xanthine oxidase, CAT—catalase.

Enzymatic Assay	CPS Activity
XO	not detected
CAT	32.74 ± 0.49% of inhibition

## Data Availability

Data available on request.
